# Exploration of *in vivo* efficacy of artemether-lumefantrine against uncomplicated *Plasmodium falciparum* malaria in under fives in Tabora region, Tanzania

**DOI:** 10.1186/1475-2875-12-60

**Published:** 2013-02-11

**Authors:** Deokary Joseph, Abdunoor M Kabanywanyi, Ruth Hulser, Zulfiqarali Premji, Omary MS Minzi, Kefas Mugittu

**Affiliations:** 1 Dar es Salaam University College of Education, PO Box 2329, Dar es Salaam, Tanzania; 2 Ifakara Health Institute, PO Box 78373, Dar es Salaam, Tanzania; 3 St Phillip’s Health Centre, PO Box 1408, Tabora, Tanzania; 4 Muhimbili University of Health and Allied Sciences, PO Box 65001, Dar es Salaam, Tanzania; 5 Ifakara Health Institute, PO Box 74, Bagamoyo, Tanzania

## Abstract

**Background:**

Tanzania adopted artemether-lumefantrine (AL) as first-line drug for uncomplicated malaria in 2006. Recently, there was an anecdotal report on high malaria recurrence rate following AL treatment in in the (urban and peri-urban), western part of Tanzania. The current report is an exploratory study to carefully and systematically assess AL efficacy in the area.

**Methods:**

Between June and August 2011, a total of 1,126 patients were screened for malaria, 33 had malaria, of which 20 patients met inclusion criteria and were enrolled and treated with standard dose of AL as recommended in the WHO protocol. Treated patients were followed up for 28 days to assess treatment responses. Before treatment (Day 0) and post-treatment (Day 7) plasma lumefantrine levels were determined to assess prior AL use and ascertain parasites exposure to adequate plasma leveles of lumefantrine, respectively.

**Results:**

The cure rate was 100%. All Day 0 plasma lumefantrine were below HPLC detectable level. The median Day 7 lumefantrine concentration was 404, (range, 189–894 ng/ml). Six out of 20 patients (30%) were gametocytaemic and all cleared gametocytes by Day 14. One patient showed an increase in gametocytes from four on Day 0 to 68, per 500 WBC on Day 2.

**Conclusion:**

Artemether lumefantrine is highly efficacious against uncomplicated *Plasmodium falciparum* malaria. The elevation of gametocytaemia despite AL treatment needs to be evaluated in a larger study.

## Background

The adoption of artemisinin-based combination therapy (ACT) in sub-Saharan Africa as first-line drug for the treatment of uncomplicated malaria [1] was largely based on experience gained in low transmission areas of Southeast Asia using artesunate-mefloquine (AS-MQ) [2]. The few studies conducted in sub-Saharan Africa prior to adoption of AS+AQ and AL recorded nearly 100% cure rate [3,4]. However, the long-term impact and extended useful therapeutic life of these drug combinations in high transmission areas remains unclear. Already increased parasite clearance half-life and reduced susceptibility to artemisinins and artesunate-mefloquine has been reported in Thai-Cambodia and Thai-Myanmar boarder [5,6]. Indeed there is now both phenotypic [7] and genetic [8] evidence of artemisinin resistance in these areas. It is hypothesized that if not controlled, artemisinin resistance might eventually spread all over the malaria-endemic world. Thus WHO recommends increased monitoring and surveillance to evaluate the threat of artemisinin resistance and identify new foci rapidly and to provide information for immediate and comprehensive containment and prevention activities. Therefore, this exploratory study was carried out as an action plan in response to reports of high recurrent rate following AL treatment in Tabora, western Tanzania (pers comm, Dr Ruth Hulser, St Phillip’s Health Centre, Tabora) in order to rule out possibility of AL resistance in the region.

## Methods

### Study site, design, sampling frame and sample size

An *in vivo* study was carried out between mid-June and mid-August 2011 at Kitete Regional Hospital and St. Philip’s and St Anne’s health facilities serving the urban and peri-urban areas of Tabora municipality. Tabora is a high-burden area and malaria transmission peaks during the rain season, i.e., February to May [9]. Children aged six to 59 months reporting to the hospital with fever or history of fever underwent physical and clinical examination by the study clinician. Inclusion and exclusion criteria are as described in the WHO protocol for high transmission areas [10]. The recruited patients were treated under supervision with a standard six doses of dispersible formulation of AL (Coartem®, Novartis) with full cream milk. The first dose was given at the health centre by a study nurse or clinician and the subsequent doses were given at home by a parent or guardian and monitored by study personnel through mobile phone call or home visits if the parent/guardian was not reachable by mobile phone. Three children vomited within 30 min of first dose and replacement dose was given and no patient vomited a replaced dose.

**Figure 1:**
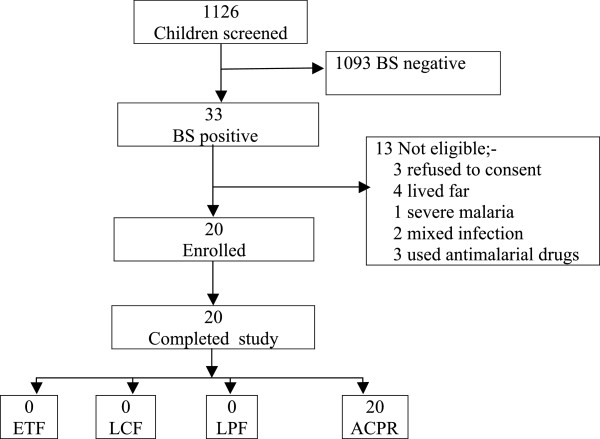
**Study profile.** Key: “Complete study” represents children who had been followed up to Day 28; ETF = early treatment failure; LCF = late clinical failure; LPF = late parasitological failure; ACPR = adequate clinical and parasitological responses; BS = blood smear.

The clinical and parasitological responses were assessed on Day 2, 3, 7, 14, 21 and 28 during the 28-day follow-up period. Parasitological responses were assessed microscopically by counting against 200 or 500 white blood cells for asexual and sexual (gametocytes), respectively. Gametocytes were not staged. Admittedly, one major limitation is that microscopy understimates the prevalence of gametocytes. Plasma lumefantrine levels were determined before treatment (Day 0) and post-treatment (Day 7). In the three months, of 1,126 children screened 33 had malaria and only 20 met the inclusion criteria and were recruited (Figure 1), following the recommendations of at least 12 patients per treatment for an exploratory study [11]. The primary efficacy endpoints were classified as: early treatment failure (ETF), late clinical failure (LCF), late parasitological failure (LPF) and adequate clinical and parasitological response (ACPR) on 28 days’ follow-up period [12]. There was no loss to follow up despite the fact that some patients came as far as 10 km away from the health facilities.

### Determination of plasma lumefantrine levels

From each recruited patient, 2 ml venous blood was collected into heparinized vacutainers on Day 0 and Day 7 for plasma drug level determination. The plasma was separated from blood cells by centrifugation, transferred into 2 ml cryovials and stored in liquid nitrogen. All plasma samples were sent to Muhimbili University College of Health and Allied Sciences (MUHAS) laboratory for lumefantrine level analysis by high performance liquid chromatographic (HPLC) method as described [12].

The study protocol was reviewed and approved by Ethical Review Committee of the Ifakara Health Institute (IHI) and MUHAS. Prior to the trial, a written consent form was signed by the parent or guardian of each participating child.

## Results

### Study profile and treatment outcome

A total number of 1,126 patients were screened from the three health facilities in Tabora region as follows: St Philip’s Health Centre: n= 663; St Anne’s Health Centre: n=345; and, Kitete Regional Hospital: n=118. The baseline characteristics of the study participants are indicated in Table 1.

**Table 1:** Baseline characteristics of the study participants

	**n**	**Minimum**	**Maximum**	**Mean**	**Std Deviation**
Age (months)	20	6.00	59.00	39.3500	18.65
Weight (Kg)	20	6.7	23.4	14.9	4.5
Temp(^0^C)	20	35.3	39.7	37.8	1.4
Par./200 wbc	20	56	3577	985	986
Parasites/μl	20	2240	143,080	39,400	39450
Hb (g/dl)	20	6.3	12.9	9.1	1.7

Twenty-four (24) out of 663 (3.6%), five out of 345 (1.4%) and four out of 118 (3.4%) patients in St Phillip’s, St Anne’s Health Centres and Ketete Hospital, respectively, were positive for malaria parasites by microscopy. Comparison of routine (before the study) and expert (during the study) malaria diagnosis was done. The routine and expert malaria microscopy results recorded in the first (50.3% positive n=163) and second half (5.1% positive n=176) of June 2011, respectively, were significantly different (p < 0.005). Only 20 patients met the inclusion criteria and were enrolled. Patients were treated and assessed for treatment outcome without loss of follow up as detailed in Figure 1. The cure rate of the drug was 100%. Table 2 shows that the mean haemoglobin was significantly increased, from 9.1 (±1.69) g/dL on Day 0 to 9.6 g/dL (±1.62) on Day 7 (p=0.048).

**Table 2:** Haemoglobin levels on Day 0 and Day 7

	**N**	**Minimum**	**Maximum**	**Mean**	**Std Deviation**
Day 0 Hb(in g/dl)	20	6.30	12.9	9.14	1.69
Day 7 Hb (g/dl)	20	6.90	12.70	9.64	1.62

### Plasma lumefantrine levels of patients

The lumefantrine plasma concentrations measured in patients before drug administration in all 20 enrolled patients were below HPLC detection level. The median Day 7 lumefantrine concentration level was 404.4 ng/ml (range 189.4-894.3 ng/ml). Only four (20%) patients had lumefantrine plasma concentration level < 280 ng/ml, and no patient had lumefantrine plasma concentration level <175 ng/ml. Lumefantrine concentration at day 7 was slightly decreased with unit increase in weight (kg) for any age but this decrease was not statistically significant (p-value = 0.445).

### Gametocyte prevalence

Table 3 shows gametocytaemia before and after AL treatment. Six of the 20 patients (30%) had gametocytes under microscopic observation on Day 0 and all were above 36 months of age. In five patients the gametocytes persisted up to Day 7 after treatment and one patient had gametocytes up to Day 14 and one had gametoctes only on Day 0. One patient showed an increase in gametocytes after treatment from four gametocytes per 500 WBC on Day 0 to 68 gametocytes per 500 WBC on Day 2 followed by a decrease on subsequent days after treatment. No gametocytes were seen microscopically after Day 14.

**Table 3:** Presence of gametocytes in six children by time

**Patient (ID)**	**Age (months)**	**Number of gametocytes per 500 WBC**						
		**Day 0**	**Day 2**	**Day3**	**Day 7**	**D ay 14**	**Day 21**	**Day 28**
1	59	7	0	0	0	0	0	0
3	48	4	68	43	5	0	0	0
4	59	145	123	85	28	0	0	0
10	50	6	4	5	2	0	0	0
16	36	13	10	7	4	0	0	0
20	59	11	3	2	2	2	0	0

## Discussion

Artemisinins are known to be highly potent anti-malarial drugs that are active against immature gametocytes [13,14], hence pivotal in reduction of malaria transmission and elimination/eradication agenda. To the best of authors’ knowledge, there is no evidence of clinical resistance to AL. However tolerance or increased parasite clearence time and reduced susceptibility to artemisinins and artesunate-mefloquine has been observed in Thai-Cambodia and Thai-Mynmar boarder [5,6]. This is the same locus where chloroquine resistance first emerged and subsequently spread all over the world. Emergence of artermisinin resistance would be disastrous for global malaria control. Therefore, regular surveillance and prompt response to any indication of compromised efficacy is required [15].

This study recorded high (100%) efficacy of AL. The reported efficacy level is more or less similar to >98% recorded in Tanzania, prior to introduction of AL [4], and elsewere in Africa [16-20]. However, in these other studies, the high efficacy rates were reached after exclusion of new infection by molecular genotyping. Therefore, it is obvious that the cause of alarm that prompted this exploratory study in Tabora was not malaria recurrence or compromised AL efficacy but malaria overdiagnosis. This practice has been reported previously elsewhere in Africa [21,22] as a cause of inappropriate malaria case managment [23]. Overdiagnosis is attributable to incompetency of laboratory workers, work overload, and pressure from patients with fever accepting malaria diagnosis [21].

In this study, none of the patients reported to have taken AL prior to hospital presentation, which was confirmed by lack of lumefantrine traces in the plasma. In addition, all patients had adequate therapautic levels of lumefantrine on Day 7. However, the Day 7 plasma lumefantrine levels showed wide variation between individuals. Indeed, it is known that the efficacy of AL combination is strongly influenced by wide variation in the pharmacokinetics of lumefantrine among individuals [24]. The maximum therapeutic cure rate is achieved when the plasma drug concentration is adequately available for at least three parasite life cycles that is equal to six days [25]. The impact of pharmacokinetic variation on selection for resistance to residual parasites in high transmission areas needs to be carefully evaluated.

Artemether-lumefantrine showed short fever and parasites clearence time and quick recovery of haemoglobin levels. Only one patient had fever up to Day 1 and neither fever nor parasitaemia was reported on Day 2, contrary to a report in central Ethiopia where fever and parasitaemia persisted to Day 3 [26]. Haemoglobin levels significantly increased on Day 7 as reported in other studies in this region, but the second haemoglobin measurement was done on Day 14 [27,28]. The complete 28 days’ follow up to all patients was achieved by using mobile phone calls to remind parents or guardians about their appointment dates and days. Mobile phone contacts might minimize loss to follow up in efficacy studies.

The Day 0 gametocyte prevalence (30%) recorded in this study is higher compared with those reported previously in Tanzania [29,30]. Furthermore, as observed elsewhere in Africa [27,31-33], no new gametocytaemic cases were observed during the follow-up period. Admittedly, this could be attributable to small sample size, which is a limitation of this study, and microscopy detection limit. Gametocytaemia persisted for seven to 14 days, (as recorded previously), against 55 days for non-ACT drugs [34].

ACT is effective against immature sequestered gametocytes [32,34,35], hence they reduce gametocyte carriage as well as gametocytes density [31,36,37]. Apparently, ACT does not affect the viability of mature gametocytes [31,36]. Consequently, persistence of mature gametocytes might still play a role on maintianing transmission cycle. Hence, combination with a strong gametocytocidal drug such as primaquine will maximize ACT usefulness.

The phenomenon of increase in gametocytaemia in this study and of sexual stage count in Nigeria [38] despite drug treatment, might be a sign of tolerance or emergence of parasites with reduced drug sensitivity in the region. Another plausible explanation for the increase in gametocytaemia is coincidence between drug administration and maturation release of mature gametocytes into the peripheral circulation. These observations of increased gametocytaemia after treatment need to be verified in a larger study.

## Conclusion

The therapeutic efficacy of AL against uncomplicated *P. falciparum* malaria was high in the study area. All patients were not exposed to AL prior to hospital presentation and levels of lumefantrine on Day 7 were therapeutically adequate but highly variable. The unusual increase in gametocytes following treatment needs be evaluated in a larger study concurrent with improvement capacity to diagnose malaria and handling of non-malarial fevers.

## Competing interests

The authors declare that they have no competing interests.

## Authors’ contributions

KM, AM and ZP designed the study. KM and DJ supervised the field work. SA and AMK and ZP supervised the clinical part. MOMS supervised the drug level analysis and pharmacokinetics. DJ was involved in drug analysis and analysed data. RH is the in charge of St Philip’s Health Centre. DJ and KM wrote the manuscript. All authors read and approved the final manuscript.
